# Robot-Assisted Partial Cystectomy in a 10-Year-Old: Managing a Rare Bladder Leiomyoma With Minimally Invasive Surgery

**DOI:** 10.7759/cureus.111576

**Published:** 2026-06-26

**Authors:** Elizabeth Chu, Benjamin H Press, Maria V Rodriguez, Andrew J Kirsch

**Affiliations:** 1 Urology, Emory University, Atlanta, USA; 2 Pediatric Urology, Children's Healthcare of Atlanta, Atlanta, USA; 3 Pediatric Urology, Georgia Urology, Atlanta, USA

**Keywords:** bladder leiomyoma, minimally invasive surgery, partial cystectomy, pediatric urology, robotic urologic surgery

## Abstract

Bladder leiomyomas are rare benign smooth muscle tumors, exceptionally uncommon in children. We report a 10-year-old boy with a one-week history of severe penile pain during urination. Imaging revealed a 4.4-cm anterior bladder wall mass initially suspected to be a urachal cyst; biopsy showed a fibroinflammatory lesion. He underwent cystoscopy and robot-assisted laparoscopic partial cystectomy with complete excision and negative margins. The postoperative course was uncomplicated, with symptom resolution. Final pathology confirmed an infarcted leiomyoma within the muscularis propria. This case highlights that robot-assisted laparoscopic partial cystectomy is a safe, feasible, minimally invasive option for definitive management in selected pediatric patients.

## Introduction

Bladder leiomyomas are benign mesenchymal tumors arising from the smooth muscle of the bladder wall. They are extremely rare in adults, accounting for less than 0.43% of all bladder tumors [1], and even more uncommon in pediatric patients. A comprehensive literature review identified six prior pediatric bladder leiomyoma cases, including reports in non-English-language journals that have been variably captured in prior reviews [2-5]. 

Clinical presentation varies but typically includes lower urinary tract symptoms such as dysuria, hematuria, urgency, frequency, incontinence, and abdominal or suprapubic pain [6]. Depending on tumor size and location, symptoms may be more severe, resulting in obstructive or irritative findings. Bladder leiomyomas are classified as endovesical (63%), intramural (7%), or extravesical (30%) based on their relationship to the bladder wall, which influences both presentation and surgical approach [7]. Complete surgical excision is the gold standard treatment and is curative with negligible recurrence risk [7,8]. Options range from transurethral resection for small endovesical lesions to partial cystectomy for larger or intramural tumors. In adults, robot-assisted approaches have been successfully described for bladder leiomyoma excision [9]. 

In pediatric urology, robotic surgery has been increasingly adopted with demonstrated feasibility and safety, yet its application to benign bladder tumors in children has not been previously reported. Reported pediatric cases have been managed using open and endoscopic surgical approaches [2-5]. 

We present a rare case of an anterior bladder leiomyoma in a 10-year-old boy treated with robot-assisted laparoscopic partial cystectomy. To our knowledge, this is the first reported pediatric case of bladder leiomyoma managed using a robotic surgical approach. This article was previously presented as a video abstract at the 2026 American Urologic Association Annual Meeting on May 17, 2026.

## Case presentation

A 10-year-old boy with autism presented to the pediatric urology clinic with a one-week history of severe penile pain during urination. Physical examination was unremarkable. Urinalysis showed trace blood. A renal and bladder ultrasound was performed, which was notable for a 4.2-cm mass at the anterior bladder wall. He then underwent a CT scan of the abdomen and pelvis (Figure 1), which demonstrated a 4.4-cm, slightly lobulated cystic structure with a peripherally enhancing thick wall causing a mass effect on the bladder. This was felt to be most consistent with an infected urachal cyst. 

**Figure 1:**
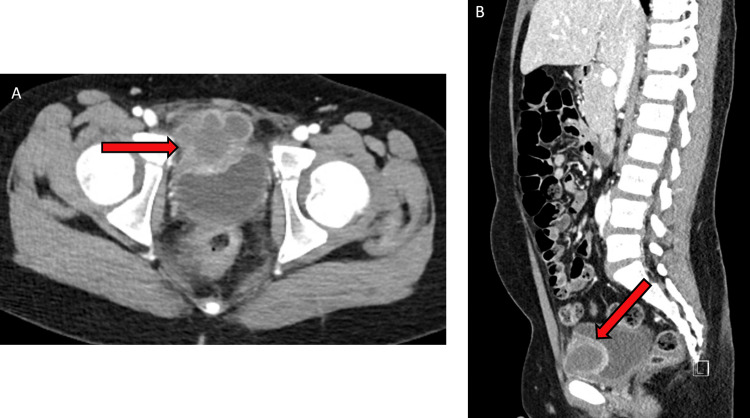
Preoperative CT scan Axial (A) and sagittal (B) views of the initial CT scan, which demonstrated a lobulated mass anterior to the bladder (red arrow).

The patient then underwent ultrasound-guided aspiration and biopsy of the presumed urachal cyst. No fluid was aspirated, and a core biopsy confirmed the lesion was solid rather than cystic. Pathology demonstrated a fibroinflammatory lesion with eosinophils but no urachal epithelium. He was therefore scheduled for surgical excision. He subsequently underwent cystoscopy, followed by robot-assisted laparoscopic partial cystectomy. No intravesical lesions were noted on cystoscopy. Once the robot was docked, the median umbilical ligaments were incised, and the bladder was mobilized from the anterior abdominal wall to develop the space of Retzius below the pubic bone. This allowed for visualization of the lobulated mass on the anterior surface of the bladder (Figure 2).

**Figure 2:**
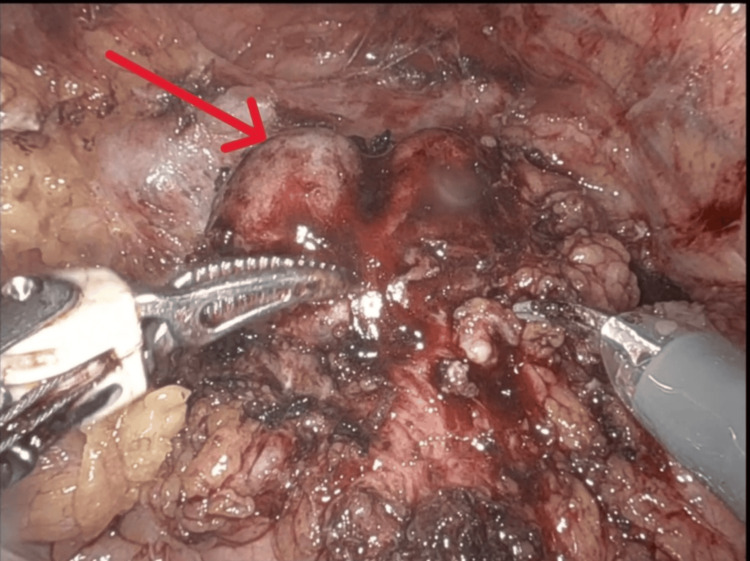
Robotic intraoperative view demonstrating the anterior bladder wall mass after mobilization from the anterior abdominal wall Following release of the bladder from the anterior abdominal wall, the anterior bladder mass (red arrow) was visualized in its entirety. It was then excised completely using a combination of electrocautery and sharp dissection.

The extravesical surface around the tumor was circumferentially marked using electrocautery, which allowed for a margin of normal tissue. The full‐thickness bladder tumor site was sharply excised with a wide margin. Intraoperative frozen section analysis demonstrated no evidence of malignancy. Surgical margins were negative. The bladder wall was closed in two layers with 3-0 Vicryl and a 3-0 V-Loc suture (Figure 3).

**Figure 3:**
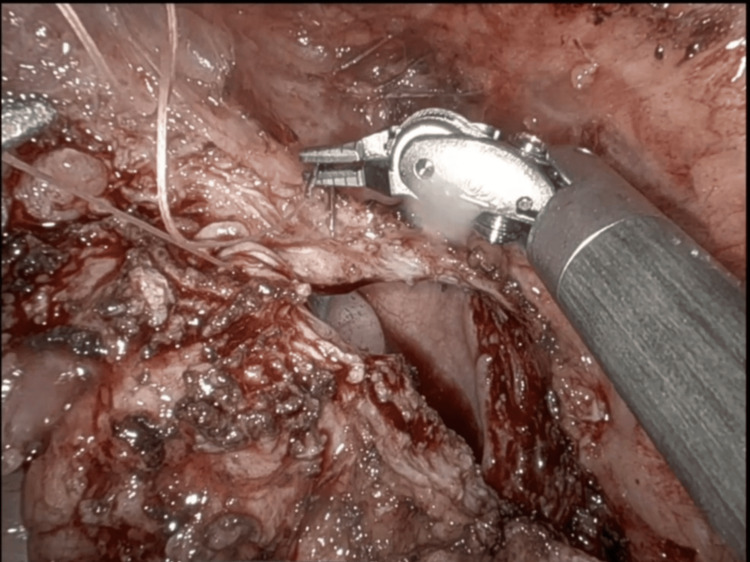
Robotic intracorporeal closure of the bladder defect First layer of bladder closure performed with 3-0 Vicryl suture.

The bladder was irrigated with sterile normal saline, confirming a watertight closure. The patient recovered uneventfully and was discharged on postoperative day 1. The catheter was removed one week postoperatively. Pathology demonstrated an extensively necrotic smooth muscle tumor within the bladder muscularis propria, consistent with an infarcted leiomyoma. Following resection of the mass, the patient has had resolution of his symptoms. After one year of follow-up, he remains asymptomatic. Renal and bladder ultrasound demonstrated normal kidneys bilaterally, without hydronephrosis, masses, or other abnormalities. The bladder was decompressed at the time of imaging, with no evidence of recurrent tumor or ureteral dilation.

## Discussion

In children, the most common bladder tumors are rhabdomyosarcoma and papillary urothelial neoplasms of low malignant potential (PUNLMP), the former malignant and the latter benign. One study reported PUNLMP as the most prevalent bladder tumor (51.3%), followed by embryonal rhabdomyosarcoma (29.3%) [10,11]. In contrast, bladder leiomyomas comprise only 0.43% of bladder tumors [1]. 

Approximately 50% of bladder leiomyoma patients present with obstructive symptoms, followed by irritative symptoms, then hematuria [9]. Our patient’s presenting symptoms of penile pain and post-void dribbling were unusual compared with previously reported cases. For example, Chen et al. described lower abdominal pain, while Mitra et al. reported hematuria, post-void abdominal pain, and intermittent fever [2,3].

Ultrasound is typically the first-line imaging modality given its sensitivity, accessibility, and low cost. Leiomyomas are usually described as homogeneous solid masses, though partially cystic-appearing lesions have been reported [12]. Further evaluation with CT or MRI allows a more accurate assessment of tumor margins. In our case, CT imaging suggested a cystic urachal lesion rather than the more typical solitary tumor protruding into the urinary bladder, complicating diagnosis [3,12]. Percutaneous biopsy, although not routinely performed in the evaluation of bladder leiomyomas, was pursued after the attempted aspiration of the presumed urachal cyst was unsuccessful because the lesion was found to be solid.

The gold standard treatment for bladder leiomyomas is complete surgical excision, which is curative with negligible recurrence risk [4]. Small endovesical tumors are typically managed with transurethral resection of bladder tumor (TURBT). Larger or more complex lesions may require open or laparoscopic excision of the tumor [9,10]. Endovesical tumors at the bladder neck or urethra are best suited for TURBT [13]. To our knowledge, no robotic approach has previously been reported in this population [2-5]. The initial report of an 11-year-old female was managed with open excision [4]. Subsequent reports included a 17-year-old male treated with transurethral resection for two small bladder lesions [14], a six-year-old male who underwent open partial cystectomy [3], another six-year-old male treated with wide local excision [2], and a 15-year-old female treated with transurethral resection [5].

Complete surgical excision remains the definitive treatment for bladder leiomyomas, with an excellent prognosis and low risk of recurrence [4]. Historically, management has ranged from transurethral resection for small endoluminal lesions to open partial cystectomy for larger or intramural tumors [9,10,13]. However, as minimally invasive surgery has advanced, laparoscopic and robotic approaches have emerged as viable alternatives, offering reduced morbidity and enhanced visualization. In adults, robot-assisted laparoscopic partial cystectomy has been increasingly utilized for benign and malignant bladder lesions due to improved dexterity, three-dimensional visualization, and the ability to perform precise intracorporeal reconstruction [9,15].

To our knowledge, this is the first case of a bladder leiomyoma in a pediatric patient managed with robotic partial cystectomy, highlighting the expanding role of robotic surgery in complex pediatric urologic reconstruction. The robotic platform provided several advantages, including superior visualization of the tumor margins, enhanced instrument articulation within the confined pediatric pelvis, and the ability to perform a meticulous bladder closure with minimal blood loss. These benefits are particularly relevant in children, in whom preserving bladder capacity and avoiding unnecessary morbidity are essential considerations.

## Conclusions

Bladder leiomyomas are exceptionally rare benign tumors in pediatric patients and may present with nonspecific symptoms and atypical imaging findings that complicate diagnosis. This case highlights the importance of maintaining a broad differential diagnosis for pediatric bladder masses, particularly when presumed urachal lesions demonstrate unusual characteristics. Complete surgical excision remains curative, and our experience demonstrates that robot-assisted laparoscopic partial cystectomy is a safe and effective minimally invasive option for benign bladder masses in carefully selected children. To our knowledge, this represents the first reported pediatric case of bladder leiomyoma managed with a robotic approach, further supporting the expanding role of minimally invasive robotic surgery in complex pediatric urologic reconstruction.

## References

[1] Hong SY, Lee TS, Kim JH (2009). Laparoscopic enucleation of a bladder leiomyoma. J Minim Invasive Gynecol.

[2] Mitra S, Joshi R, Gupta K, Mahajan JK (2019). Not all pediatric bladder masses are rhabdomyosarcoma: report of a rare case with review of the literature. J Pediatr Hematol Oncol.

[3] Chen H, Niu ZB, Yang Y (2012). Bladder leiomyoma in a 6-year-old boy. Urology.

[4] Mutchler RW Jr, Gorder JL (1972). Leiomyoma of the bladder in a child. Br J Radiol.

[5] Genc YE, Ulker ND, Toper MH, Yucel S, Sener TE (2024). Bladder leiomyoma in a 15-year-old female patient: a case report. Cureus.

[6] Shelmerdine SC, Lorenzo AJ, Gupta AA, Chavhan GB (2017). Pearls and pitfalls in diagnosing pediatric urinary bladder masses. Radiographics.

[7] Goluboff ET, O'Toole K, Sawczuk IS (1994). Leiomyoma of bladder: report of case and review of literature. Urology.

[8] Park JW, Jeong BC, Seo SI, Jeon SS, Kwon GY, Lee HM (2010). Leiomyoma of the urinary bladder: a series of nine cases and review of the literature. Urology.

[9] Thiel DD, Williams BF, Krishna M, Leroy TJ, Igel TC (2009). Robot-assisted laparoscopic excision of bladder wall leiomyoma. J Endourol.

[10] Feng S, Yang Y, Yu Z, Xiong Q, Bi Y (2023). Survival and analysis of prognostic factors for bladder malignancies in children and adolescents: a population-based study. Urology.

[11] Shumaker AD, Harel M, Gitlin J (2022). Pediatric bladder tumors: a ten-year retrospective analysis. Urology.

[12] Khater N, Sakr G (2013). Bladder leiomyoma: presentation, evaluation and treatment. Arab J Urol.

[13] Kabalin JN, Freiha FS, Niebel JD (1990). Leiomyoma of bladder. Report of 2 cases and demonstration of ultrasonic appearance. Urology.

[14] Moyano Calvo JL, Maqueda Marín Mde L, Dávalos Casanova G (2005). Bladder leiomyoma in a 17-year-old male patient (Article in Spanish). Arch Esp Urol.

[15] Khaitan A, Seth A, Dinda AK, Singh I, Talwar M, Bandhu S (2002). Transurethral resection versus open surgery for leiomyoma of urinary bladder--a report of 2 cases. Int Urogynecol J Pelvic Floor Dysfunct.

